# Aurora kinase B is important for antiestrogen resistant cell growth and a potential biomarker for tamoxifen resistant breast cancer

**DOI:** 10.1186/s12885-015-1210-4

**Published:** 2015-04-08

**Authors:** Sarah L Larsen, Christina W Yde, Anne-Vibeke Laenkholm, Birgitte B Rasmussen, Anne Katrine Duun-Henriksen, Martin Bak, Anne E Lykkesfeldt, Tove Kirkegaard

**Affiliations:** 1 Breast Cancer Group, Unit of Cell Death and Metabolism, Danish Cancer Society Research Center, Copenhagen, Denmark; 2 Department of Pathology, Slagelse Hospital, Slagelse, Denmark; 3 Department of Pathology, Herlev Hospital, Herlev, Denmark; 4 Statistics, Bioinformatics and Registry, Danish Cancer Society Research Center, Copenhagen, Denmark; 5 Department of Clinical Pathology, Odense University Hospital, Odense, Denmark; 6 Present address: Department of Surgery, Køge Hospital, Køge, Denmark

**Keywords:** Antiestrogen resistance, Breast cancer, Fulvestrant, Tamoxifen, Barasertib, Aurora kinase B

## Abstract

**Background:**

Resistance to antiestrogen therapy is a major clinical challenge in the treatment of estrogen receptor α (ER)-positive breast cancer. The aim of the study was to explore the growth promoting pathways of antiestrogen resistant breast cancer cells to identify biomarkers and novel treatment targets.

**Methods:**

Antiestrogen sensitive and resistant T47D breast cancer cell lines were used as model systems. Parental and fulvestrant resistant cell lines were subjected to a kinase inhibitor library. Kinase inhibitors preferentially targeting growth of fulvestrant resistant cells were identified and the growth inhibitory effect verified by dose–response cell growth experiments. Protein expression and phosphorylation were investigated by western blot analysis. Cell cycle phase distribution and cell death were analyzed by flow cytometry. To evaluate Aurora kinase B as a biomarker for endocrine resistance, immunohistochemistry was performed on archival primary tumor tissue from breast cancer patients who have received adjuvant endocrine treatment with tamoxifen.

**Results:**

The selective Aurora kinase B inhibitor barasertib was identified to preferentially inhibit growth of fulvestrant resistant T47D breast cancer cell lines. Compared with parental cells, phosphorylation of Aurora kinase B was higher in the fulvestrant resistant T47D cells. Barasertib induced degradation of Aurora kinase B, caused mitotic errors, and induced apoptotic cell death as measured by accumulation of SubG1 cells and PARP cleavage in the fulvestrant resistant cells. Barasertib also exerted preferential growth inhibition of tamoxifen resistant T47D cell lines. Finally, high percentage of Aurora kinase B positive tumor cells was significantly associated with reduced disease-free and overall survival in 261 ER-positive breast cancer patients, who have received tamoxifen as first-line adjuvant endocrine treatment.

**Conclusions:**

Our results indicate that Aurora kinase B is a driving factor for growth of antiestrogen resistant T47D breast cancer cell lines, and a biomarker for reduced benefit of tamoxifen treatment. Thus, inhibition of Aurora kinase B, e.g. with the highly selective kinase inhibitor barasertib, could be a candidate new treatment for breast cancer patients with acquired resistance to antiestrogens.

## Background

The selective estrogen-receptor α (ER) modulator, tamoxifen, is the recommended first-line adjuvant endocrine therapy for premenopausal women with ER-positive breast cancer, whereas postmenopausal women with ER-positive breast cancer will be offered an aromatase inhibitor. Although many patients benefit from the treatment, *de novo* or acquired resistance occurs in approximately 30% of the patients, and is therefore a major clinical challenge [1,2]. Following relapse, many patients will benefit from treatment with the pure antiestrogen fulvestrant, a selective ER down regulator, which induces degradation of ER upon binding and subsequently abolishes ER signaling [3,4]. However, in spite of initial response, almost all patients with advanced disease eventually develop resistance against antiestrogen therapy [1,3,5-7].

Cell model systems are valuable tools to study the molecular mechanisms for endocrine resistant breast cancer. We have developed *in vitro* cell culture models based on the ER-positive and estrogen responsive human breast cancer cell lines MCF-7 and T47D [8-11]. In line with other studies, we have shown that growth of breast cancer cell lines can switch from being ER-driven to being mediated by the HER receptors upon acquisition of resistance [12-18]. HER2 gene amplification or protein over expression in breast cancer is associated with a significantly shorter time to relapse, poor survival and reduced sensitivity to endocrine therapy [19-21]. We have previously shown that the expression of HER2 was increased in the T47D-derived fulvestrant resistant cell lines compared with the parental antiestrogen sensitive T47D breast cancer cells. However, resistant cell growth was not preferentially inhibited by knockdown of HER2 or by inhibition of HER receptor activity [11]. These findings indicate that HER signaling presumably does not account for all cases of breast cancer resistance, emphasizing the need for continued investigations of the resistance mechanisms.

Tumor expansion depends on continued growth of tumor cells through mitotic cell division. A key mitotic regulator is the chromosomal passenger complex (CPC), composed of the catalytic component Aurora kinase B and the three regulatory and targeting components; inner centromere protein (INCENP), survivin and borealin. CPC is important for chromosome condensation, correction of erroneous kinetochore-microtubule attachments, activation of the spindle-assembly checkpoint and cytokinesis [22]. The function of Aurora kinase B is linked to chromatin modification in relation to phosphorylation of histone H3 at Ser10 [23]. The expression of Aurora kinase B is cell cycle regulated and the kinase is activated upon binding to INCENP, which is both a substrate and a positive regulator of Aurora kinase B [24,25]. Over expression of Aurora kinase B is evident in a range of primary cancers, such as prostate, head and neck, colon and thyroid cancers, and is associated with clinical aggressiveness [26,27].

To explore the molecular mechanisms driving antiestrogen resistant cell growth, we have utilized a large kinase inhibitor library comprising 195 kinase inhibitors on parental and fulvestrant resistant T47D breast cancer cell lines. We identified Aurora kinase B as a putative novel therapeutic target in fulvestrant and tamoxifen resistant breast cancer cells, and further explored its role in signaling and growth of fulvestrant resistant T47D cell lines by using the selective Aurora kinase B inhibitors, barasertib and hesperadin. Moreover, we investigated the clinical relevance of Aurora kinase B expression in primary tumors from breast cancer patients receiving tamoxifen as first-line adjuvant endocrine therapy.

## Methods

### Cell lines, culture condition and reagents

The T47D cell line was originally obtained from the Human Cell Culture Bank (Mason Research Institute, Rockville, MD, USA) and maintained as previously described [11]. The fulvestrant resistant cell lines; T47D/182^R^-1 (182^R^-1) and T47D/182^R^-2 (182^R^-2) were established from T47D grown with 5% fetal calf serum (FCS) and long term treated with 100 nM fulvestrant (Tocris, Avonmouth, Bristol, UK) as described in [11]. To enable ER-mediated growth inhibition by tamoxifen, the T47D cell line was adapted to grow in medium (RPMI, 8 μg/ml insulin and 2 mM glutamax) supplemented with only 2% FCS (T47D/S2). This cell line was used for establishment of the tamoxifen resistant cell lines T47D/TR-1 (TR-1) and T47D/TR-2 (TR-2) [28]. The fulvestrant and tamoxifen resistant cell lines were maintained in the same growth medium as their parental T47D cell lines plus 100 nM fulvestrant or 1 μM tamoxifen (Sigma-Aldrich, St. Louis, MO, USA), respectively. For experiments, 2.5 × 10^5^ U penicillin and 31.25 μg/l streptomycin (Gibco, Life Technologies, Carlsbad, CA, USA) were added to the growth medium. Barasertib was purchased from Selleck Chemicals (Houston, TX, USA). Stock solutions of 10^−3^ M fulvestrant were dissolved in 96% ethanol, whereas stock solutions of 10 mM barasertib were dissolved in dimethyl sulfoxide (DMSO).

### Kinase inhibitor screen

The kinase inhibitor library comprising 195 different kinase inhibitors was purchased from Selleck Chemicals and the experiment was performed as previously described [28]. In brief, cells were seeded in triplicate in 96-well plates in their standard growth medium and allowed to adhere for 2 days before 5 days treatment with 1 μM of the kinase inhibitors. Vehicle-treated (0.1% DMSO) controls (6–10 wells/plate) were included in each plate. Cell viability was assayed using CellTiter-Glo Luminescent Cell Viability Assay (Promega, Madison, WI, USA) and luminescence was measured using Varioscan Flash platereader (Thermo Fisher Scientific, Waltham, MA, USA).

### Cell growth assays

Dose–response growth experiments were performed in 96-well plates. Cells were seeded in their standard growth medium and allowed to adhere for 2 days before 5 days treatment with barasertib or JNJ-7706621 (Selleck Chemicals) at indicated concentrations. Cell number was determined using a crystal violet colorimetric assay as described previously [29]. All experiments were performed at least twice with similar results. Data represent mean values of 6 wells ± SD from one representative experiment.

### Western blot analysis

To investigate the effect of barasertib on protein expression and phosphorylation of Aurora kinase A, Aurora kinase B and INCENP, as well as PARP cleavage, parental and fulvestrant resistant T47D cell lines were treated for 4–96 hours with 50 nM barasertib (Selleck Chemicals). Cell lysis and western blot analyses were performed as previously described [11]. Antibodies targeting the following proteins were used: Aurora kinase B (1:1000, #AJ1069a, Nordic Biosite, Copenhagen, Denmark), pThr288-Aurora kinase A/pThr232-Aurora kinase B/pThr198-Aurora C (1:1000, #2914, Cell Signaling Technology, Danvers, MA, USA), INCENP (1:2000, #ab12183, Abcam, Cambridge, MA, USA), Hsp70 (1:500,000, #MS-482-PO, Thermo Fisher Scientific), and PARP1 (1:1400, #6639GR, BD, Franklin Lakes, NJ, USA). All experiments were performed using at least two independent sets of lysates with similar results. Quantification was performed using Image J. The protein expression level of the specific proteins was measured relative to the respective Hsp70 loading control. The level in parental and untreated cells was set to 1.0.

### Flow cytometry

For cell cycle analysis, cells were fixed in 70% ethanol and incubated for 30 min with 20 μg/ml propidium iodide (Sigma-Aldrich, Copenhagen, Denmark) and 40 μg/ml RNase A (Roche, Basel, Switzerland) [30]. To detect the fraction of phospho-Histone H3 Ser10 positive cells, cells were fixed in 2% formaldehyde (37°C, 10 min), permeabilized in 90% ethanol (−20°C, overnight) and incubated 1 hour at 37°C with AlexaFluor488-conjugated phospho-S10-Histone H3 antibody (1:50, #3465, Cell Signaling Technology) before staining with 20 μg/ml propidium iodide (Sigma-Aldrich). Cell death was measured utilizing a SYTOX green assay, as previously described [31]. Briefly, cells were incubated with 0.5 μmol/L SYTOX green nucleic acid stain (Life Technologies) (37°C, 15 min), harvested in AccuMax (Thermo Fisher Scientific) and pooled with cells from the growth medium. Samples were subsequently resuspended in 1% FBS in PBS and kept on ice. All samples were analyzed using FACSort flow cytometer and CellQuest Pro (BD).

### Hoechst stain and fluorescence imaging

T47D, 182^R^-1 and 182^R^-2 cells were seeded in SlideFlask Chambers and treated with 0.1% DMSO (control) or 50 nM barasertib. The cells were fixed in 4% formaldehyde, permeabilized by 0.2% Triton X-100, stained with Hoechst 33342 (Life Technologies, 1:40,000) and mounted using Fluorescence mounting medium (Dako, Glostrup, Denmark). Pictures were captured using Zeiss AX10 Imager A2 microscope (Carl Zeiss Microscopy, LLC, Thornwood, NY, USA).

### Patients

The cohort included 268 high-risk ER-positive postmenopausal breast cancer patients diagnosed between 1989 and 2001. The patients had received tamoxifen as first-line adjuvant endocrine treatment according to the guidelines from the Danish Breast Cancer Cooperative Group (DBCG) [32]. The standard clinico-pathological parameters have previously been published [33]. The biomarker study was approved by the local ethics committee for Region South Denmark, S-VF-20040064, the Ethical Committee waived the requirement for informed consent from the participants.

### Immunohistochemistry (IHC)

IHC was conducted on tissue microarrays (TMAs) using a standard immunoperoxidase procedure [33]. In brief, TMA sections, comprising two 2 mm cores from each patient, were dewaxed and rehydrated through graded ethanol. Antigen retrieval was performed by heat-induced epitope retrieval (microwaving) for 15 minutes in 10 mM Tris, 0.5 mM EDTA, pH 9. Endogenous peroxidase activity was quenched by 3% hydrogen peroxide and non-specific binding blocked by Serum-free protein block (Dako). Aurora kinase B antibody (1:500, #AJ1069a, Nordic Biosite) was applied over night at 4°C. EnVision (Dako) was used for signal amplification and positive staining was visualized using 3.3-diaminobenzidine tetrahydrochloride (DAB; Dako). Nuclei were counterstained with haematoxylin before mounting in Pertex (Histolab, Göteborg, Sweeden). Aurora kinase B expression was scored as percentage positive tumor cells, blindly and without reference to the patient history.

### Statistics

In the kinase inhibitor screen, one-tailed Student’s *t-*test was performed on triplicate values comparing the growth inhibitory effect in parental and resistant cell lines. In the remaining experiments, group comparisons were done using a two-tailed *t-*test with Bonferroni adjusted *p*-values for multiple testing. In the biomarker study, uni- and multivariate analyses were performed. The multivariate analysis included tumor grade, size, nodal status and age as standard covariates. Kaplan-Meier life tables with log-rank testing were generated to assess the association between the percentage of Aurora kinase B positive tumor cells, and disease-free and overall survival. The statistical analysis on the clinical data was performed in R version 3.0.1, with the R package “rms”. For all experiments, P < 0.05 were considered statistically significant.

## Results

### Kinase inhibitor screen identifies barasertib as a preferential growth inhibitor of fulvestrant resistant cells

**Figure 1:**
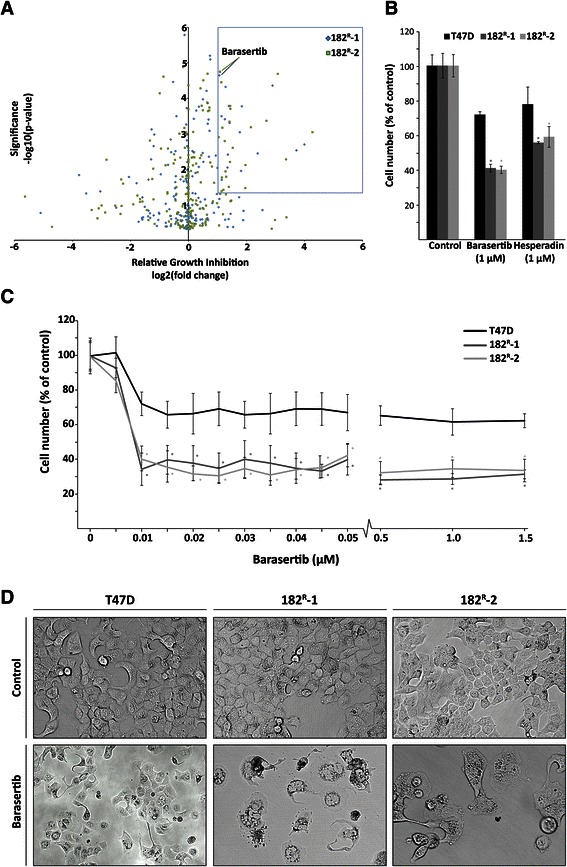
**The kinase inhibitor barasertib induces preferential growth inhibition of fulvestrant resistant cell lines. A**. Parental (T47D) and fulvestrant resistant (182^R^-1 and 182^R^-2) cell lines were treated for 5 days with a kinase inhibitor library containing 195 different kinase inhibitors (1 μM). Cell number was assessed by a CellTiter-Glo Luminescent Cell Viability Assay. In the generated volcano plot, the box indicates kinase inhibitors with more than two-fold greater growth inhibition of the fulvestrant resistant cells (182^R^-1 and 182^R^-2) compared to the parental T47D cells (P < 0.05). **B**. Mean cell numbers of parental and resistant cells treated with barasertib (1 μM) or Hesperadin (1 μM) shown as percent of untreated control. The results are from the kinase inhibitor screen. **C**. Parental and fulvestrant resistant cells treated for 5 days with the indicated concentrations of barasertib. Cell number was determined by a crystal violet colorimetric assay and expressed as percent of untreated control. The experiments were performed twice with six sample replicates. Representative experiments with mean ± SD are shown. **D**. Representative pictures of parental and resistant cells treated for 5 days with barasertib (50 nM μM) or DMSO (control). *P < 0.05 for barasertib treated samples vs. control.

To identify kinases causally involved in fulvestrant resistance, parental and fulvestrant resistant (182^R^-1 and 182^R^-2) T47D cell lines were subjected to a kinase inhibitor library comprising 195 inhibitors each targeting one or more different protein kinases. The results from the screen are shown in a volcano plot displaying, for each of the kinase inhibitors, statistical significance (P < 0.05) versus fold change in relative growth inhibition between fulvestrant resistant and parental cell lines (Figure 1A). We identified inhibitors which preferentially targeted growth of both resistant cell lines with a statistical significant growth inhibition which was at least two-fold higher than the inhibition of the parental cells. The majority of the identified kinase inhibitors which fulfilled these criteria targeted the Aurora protein kinase family, whereas no inhibitors were found to target the HER receptors or their downstream signaling molecules Akt or ERK (Table 1). Noteworthy, the specific Aurora kinase B inhibitor barasertib exerted similar growth inhibitory effect on the fulvestrant resistant cell lines as the Aurora kinase inhibitors targeting both Aurora kinase A and B, indicating that Aurora kinase B is the most important Aurora kinase in fulvestrant resistant T47D cell lines. Therefore, the highly selective Aurora kinase B inhibitor barasertib was explored further. Compared to untreated controls, barasertib (1 μM) inhibited growth of fulvestrant resistant cell lines by 60% whereas parental T47D cell growth was inhibited by only 30% (Figure 1B). Another specific Aurora kinase B inhibitor, hesperadin, also induced a preferential growth inhibition of the fulvestrant resistant cell lines in the kinase inhibitor screen, however, with a less than two-fold growth inhibition of the resistant cells compared with the parental cell line (Figure 1B).

**Table 1:** **Inhibitors identified in the kinase inhibitor screen**

Inhibitor	Inhibitory Effect ± SD (%)			Target(s)
	T47D	182^R^-1	182^R^-2	
**Barasertib**	28.2 ± 1.7	58.9 ± 2.2	59.9 ± 2.1	Aurora B
**PHA-680632**	23.5 ± 7.4	62.8 ± 2.7	56.1 ± 5.0	Aurora A/B/C
**SNS-314 Mesylate**	19.4 ± 1.5	47.0 ± 2.6	52.8 ± 5.1	Aurora A/B/C
**ZM-447439**	24.2 ± 6.1	57.7 ± 1.8	65.6 ± 1.5	Aurora A/B/C
**CCT129202**	20.1 ± 13.2	44.5 ± 4.8	48.8 ± 4.3	Aurora A/B/C
**AMG 900**	25.3 ± 3.0	55.8 ± 4.5	55.5 ± 0.6	Aurora A/B/C
**JNJ-7706621**	1.7 ± 5.7	27.5 ± 4.9	33.4 ± 4.8	Aurora A/B, cyclin A/CDK2, cyclin E/CDK2, cyclin B/CDK1
**AT9283**	6.2 ± 3.6	46.4 ± 2.5	52.6 ± 2.1	Aurora A/B, JAK2/3 and Abl
**ENMD-2076**	24.3 ± 16.5	61.8 ± 2.8	65.7 ± 1.5	Aurora A/B, Flt3/4, SFKs and VEGFR2
**AZD7762**	8.6 ± 10.3	30.3 ± 1.1	33.8 ± 2.8	Chk1/2
**CX-4945**	8.2 ± 2.1	27.9 ± 1.7	21.8 ± 1.7	CK2
**PF-562271**	1.4 ± 4.5	19.0 ± 3.5	14.8 ± 2.5	FAK and Pyk2
**Nintedanib**	11.8 ± 3.0	42.2 ± 3.5	33.9 ± 6.2	FGFR, VEGFR, PDGRF and SFKs
**Dasatinib**	18.0 ± 3.5	47.1 ± 2.7	37.0 ± 3.4	SFKs, Abl and c-Kit
**TG100-115**	5.0 ± 1.5	12.9 ± 2.3	13.2 ± 5.3	PI3Kγ/δ

The growth inhibitory effect of the kinase inhibitors compared with untreated cells ± standard deviation (SD). *Abbreviations:* Abelson *(Abl)*, Casein kinase *(CK)*, checkpoint kinase *(Chk)*, cyclin-dependent kinases *(CDK)*, fibroblast growth factor receptor *(FGFR)*, Fms-like Tyrosine Kinase *(Flt)*, focal adhesion kinase *(FAK)*, janus kinase *(JAK)*, platelet-derived growth factor receptor *(PDGFR)*, proline-rich tyrosine kinase *(Pyk)*, Src Family kinases *(SFKs)*, vascular endothelial growth factor receptor *(VEGFR)*, and phosphoinositide 3-kinase *(PI3K)*.

To our knowledge, Aurora kinase B has not previously been described to be involved in growth of antiestrogen resistant breast cancer cells. Dose–response growth experiments were conducted with increasing concentrations of barasertib (5 nM-1.5 μM), resulting in a statistical significant 70% growth inhibition of the resistant cells from 10 nM compared to only 30-40% growth inhibition of the parental T47D cells (Figure 1C). The results confirmed the preferential growth inhibition of barasertib observed in the kinase inhibitor screen (Figure 1B) and showed that the maximal growth inhibition of parental and fulvestrant resistant cell lines was obtained with only 10 nM barasertib (Figure 1C). The morphology of parental and fulvestrant resistant T47D cell lines upon five days treatment with barasertib (50 nM) revealed substantial differences between parental and resistant cell lines (Figure 1D). The morphology of parental T47D cells was only slightly affected by barasertib and the cells remained attached to the surface. In contrast, the morphology of the fulvestrant resistant cells was severely changed showing increased size of detaching apoptotic-like cells and reduced cell number.

### Fulvestrant resistant cell lines display increased Aurora kinase B phosphorylation, which is abolished by barasertib

**Figure 2:**
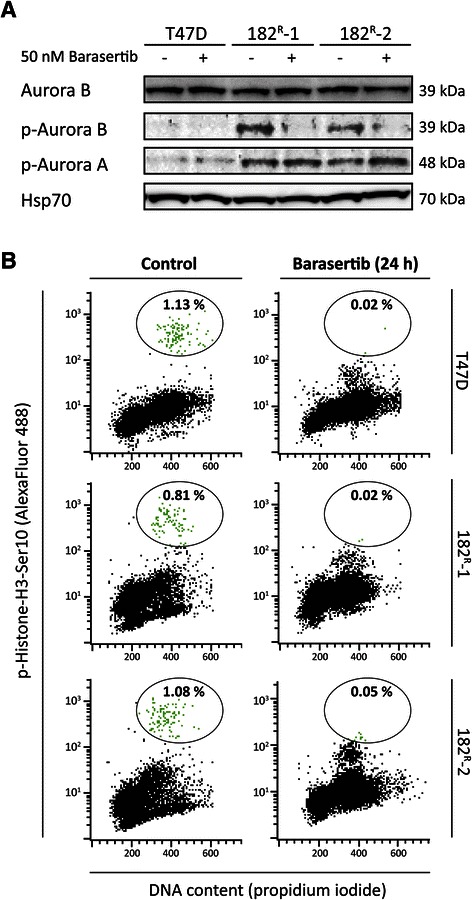
**Fulvestrant resistant cell lines exhibit increased Aurora kinase B phosphorylation, and barasertib abolishes phosphorylation of Aurora kinase B and Histone-H3. A**. Western blots showing total and phosphorylated (p) form of Aurora kinase B (Thr^232^) and Aurora kinase A (Thr^288^) in lysates from parental (T47D) and fulvestrant resistant (182^R^-1 and 182^R^-2) cells treated with barasertib (50 nM) or DMSO (control) for 4 hours. Heat shock protein 70 (Hsp70) was used as loading control. **B**. Parental and resistant cells were treated with barasertib (50 nM) or DMSO (control) for 24 hours before the cells were fixed and stained with phospho-Histone-H3 Ser10 antibody and propidium iodide prior analysis and flow cytometry performed using a FACsort flow cytometer. M-phase phospho-Histone-H3 Ser10 positive cells are encircled and the bold numbers indicate percentage of positive cells in each sample. Representative experiments are shown.

To investigate the expression and phosphorylation level of Aurora kinase B in parental and fulvestrant resistant T47D cell lines, western blot analysis was performed. Comparable Aurora kinase B expression was seen in parental and resistant cell lines, whereas phosphorylation of both Aurora kinase A and B was increased in the fulvestrant resistant cell lines compared with the parental T47D cells. Treatment with barasertib (50 nM) for 4 hours resulted in undetectable level of phosphorylated Aurora kinase B in the resistant cells, but did not have any effect on the level of phosphorylated Aurora kinase A. Only very low levels of phosphorylated Aurora kinase A and B were seen in the parental cells, and no effect of treatment with barasertib was observed (Figure 2A). Additionally, FACS analysis revealed that the percentage of cells with phosphorylated mitosis-specific histone H3, a downstream target of Aurora kinase B [34], was reduced in both parental and fulvestrant resistant cell lines upon treatment with barasertib (50 nM) for 24 hours (Figure 2B). Collectively, these data support that barasertib selectively targets Aurora kinase B.

### Barasertib induces degradation of Aurora kinase B and dephosphorylation of INCENP

**Figure 3:**
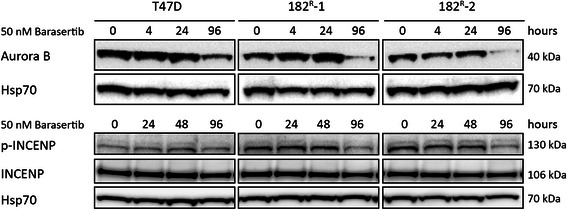
**Barasertib inhibits expression of Aurora kinase B and phosphorylation of INCENP.** Western blots showing protein expression of Aurora kinase B, INCENP and phosphorylated INCENP (p-INCENP) in lysates from parental (T47D) and fulvestrant resistant (182^R^-1 and 182^R^-2) cells treated with barasertib (50 nM) or DMSO (control) for the indicated time periods (4–96 hours). Heat shock protein 70 (Hsp70) was used as loading control.

To further explore the expression and function of Aurora kinase B in T47D breast cancer cell lines, parental and resistant cells were treated with barasertib (50 nM) for 4–96 hours. As seen in Figure 3, the expression of Aurora kinase B was reduced to 52%, 36% and 22% in parental cells, 182^R^-1 and 182^R^-2 cells, respectively, upon 96 hours treatment with barasertib. This is presumably due to degradation of Aurora kinase B as described by Gully et al. [35]. Compared with parental T47D cells, the level of the phosphorylated mitotic form of INCENP was increased by 1.25-fold and 2.25-fold in 182^R^-1 and 182^R^-2, respectively (Figure 3), whereas the level of un-phosphorylated interphase INCENP, which moves faster through the gel than phosphorylated INCENP [36], was similar in parental and resistant cell lines. Treatment with barasertib (50 nM) for 96 hours had no effect on the level of phosphorylated INCENP in the parental cells, but the levels were reduced to 49% and 42% in 182^R^-1 and 182^R^-2 cells, respectively (Figure 3).

### Barasertib induces mitotic errors and affects cell cycle phase distribution

**Figure 4:**
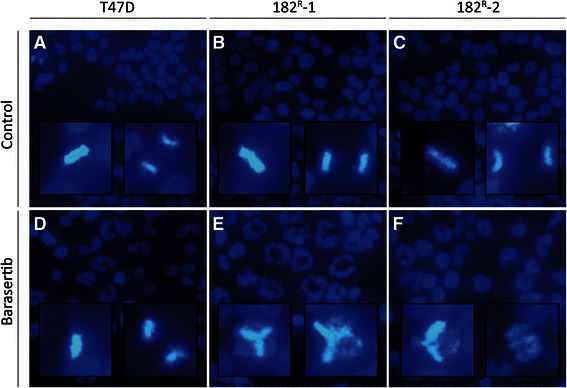
**Barasertib prevents chromosome alignment in fulvestrant resistant cell lines.** Fluorescence microscopy of Hoechst stained **(AandD)** parental, **(BandE)** 182^R^-1 and **(CandF)** 182^R^-2 T47D cells treated for 42 hours with DMSO (control; **A**-**C**) or barasertib (50 nM; **D**-**F**). Inserts show higher-magnification images of dividing cells.The experiment was repeated twice and representative images are shown.

**Figure 5:**
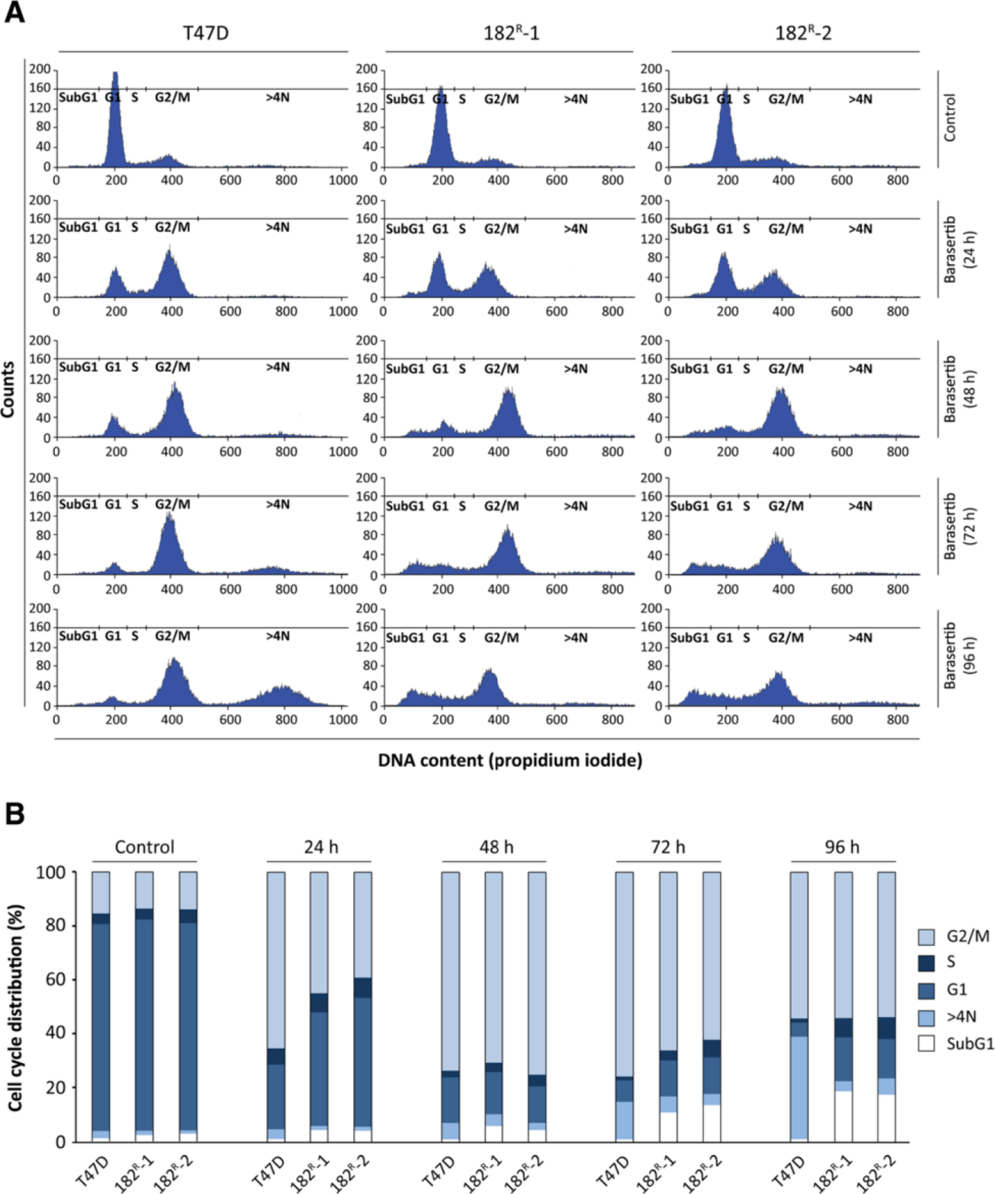
**Barasertib causes growth arrest in the G2/M cells cycle phase. A**. Parental (T47D) and fulvestrant resistant (182^R^-1 and 182^R^-1) cells treated with barasertib (50 nM) or DMSO (control) for 24–96 hours and subsequently stained with propidium iodide. Cell cycle phase distribution in the following phases are shown: G_1_ phase, S phase, G_2_/M phase, SubG1 and >4N (polyploid cells). **B**. Distribution of cells in G_2_/M, S, G_1_ and SubG1 phases and cells with DNA content above 4N are calculated by quantification of the phase fractions seen in **A**. Duration of barasertib treatment is indicated. Two individual experiments were performed and representative results are shown.

Aurora kinase B is important for correct cell cycle progression and plays a key role in the maintenance of normal ploidy level during cell division [25]. To investigate whether treatment with barasertib had an impact on chromosome segregation and cell division, cell nuclei were stained with Hoechst (Figure 4). Only a minor effect on chromosome alignment in the mitotic metaphase plane was seen in parental T47D cells treated with barasertib (Figure 4D). In contrast, in the fulvestrant resistant cell lines, barasertib had a severe effect on chromosome alignment and segregation, and no dividing sister chromatids could be observed (Figure 4E,F). Cell cycle analysis was therefore performed to investigate the effect of barasertib on cell cycle phase distribution. Parental and resistant T47D cells were treated with barasertib (50 nM) for 24–96 hours prior to staining with the nucleic acid dye propidium iodide. Histograms presenting the cell cycle phase distribution following treatment with barasertib are shown in Figure 5A. When quantified, we found that barasertib induced a shift in cell cycle phase distribution for both parental and resistant cell lines (Figure 5B). Treatment with barasertib for up to 48 hours induced accumulation of both parental and fulvestrant resistant cells in the G2/M phase with a concomitant decrease in the fraction of G1 cells. After 72 hours and in particular 96 hours, parental cells with DNA content greater than 4 N was accumulating. In fulvestrant resistant cell lines, 72 and 96 hours treatment resulted in an increase in cells with DNA content less than 2N (subG1) corresponding to dead cells (Figure 5B).

### Barasertib induces cell death of fulvestrant resistant cell lines

**Figure 6:**
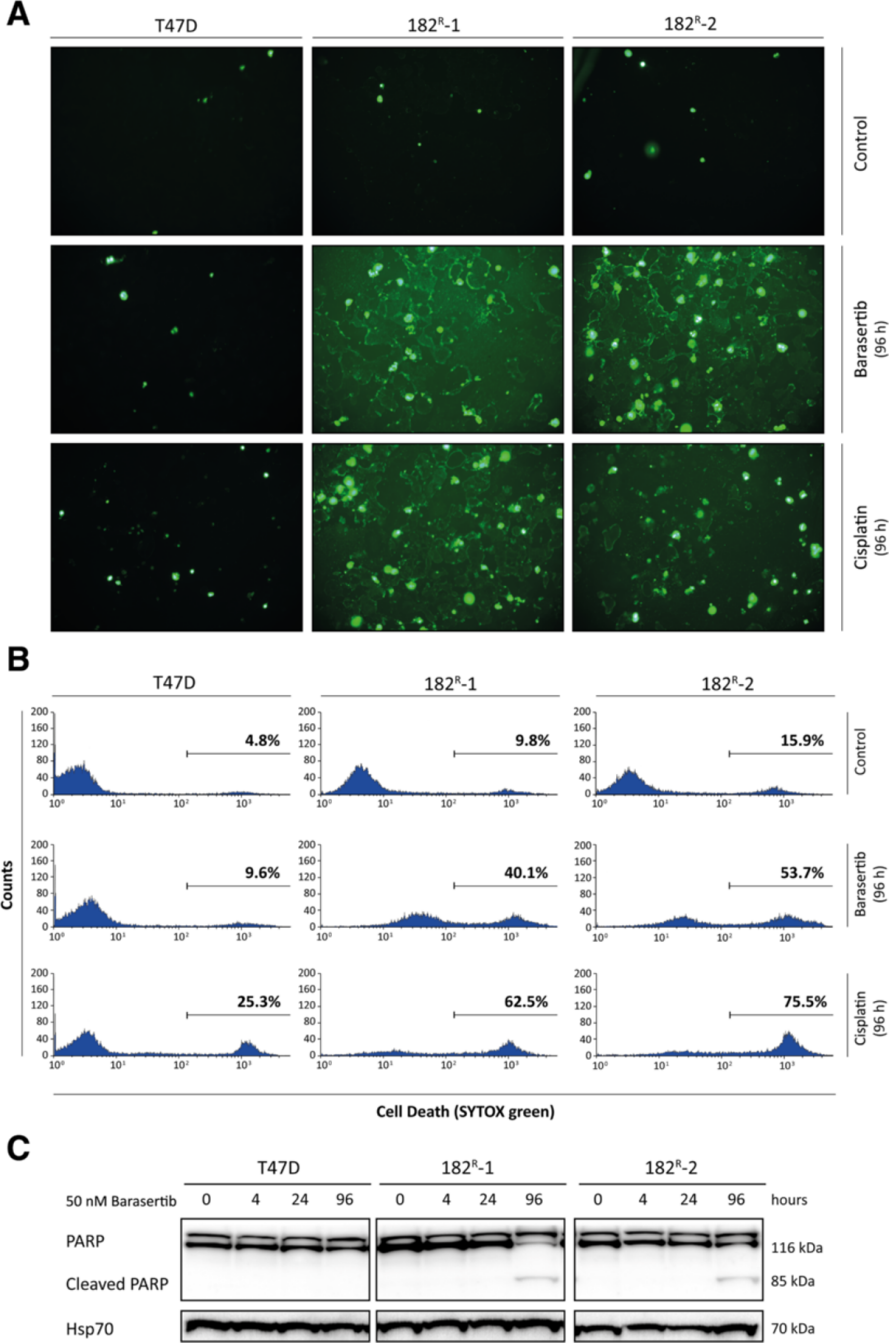
**Barasertib induces apoptotic cell death of fulvestrant resistant cell lines. A-B**. Parental (T47D) and fulvestrant resistant (182^R^-1 and 182^R^-2) cells were treated with barasertib (50 nM), cisplatin (20 μM) or DMSO (control) for 96 hours, stained with SYTOX green and analyzed by fluorescence microscopy and flow cytometry. Percentages of dead (SYTOX green positive) cells are indicated in bold **C**. Western blots showing total and cleaved form of PARP in lysates from T47D, 182^R^-1 and 182^R^-2 cells treated with barasertib (50 nM) or DMSO (control) for 4–96 hours. Heat shock protein 70 (Hsp70) was used as loading control. Two individual experiments were performed and data from one representative experiment are shown.

The large proportion of SubG1 cells in the resistant cell lines indicated induction of apoptosis upon treatment with barasertib. We therefore conducted a SYTOX green assay to further examine the effect of barasertib on cell death in parental and fulvestrant resistant cells. In the experiment, cisplatin was used as a positive control for induction of cell death (Figure 6A, B). A large number of SYTOX green-positive cells were observed in the two fulvestrant resistant cell lines treated for 96 hours with barasertib (50 nM) or cisplatin (20 μM) (Figure 6A, B). SYTOX green-positive cells were quantified by flow cytometry showing increased percentage of dead cells (bold numbers) in untreated 182^R^-1 and 182^R^-2 (9.8% and 15.9%, respectively) compared with parental T47D cells (4.8%) (Figure 6B). Only a two-fold increase in percentage of dead cells to 9.6% was detected in the parental cell line upon treatment with barasertib (50 nM) for 96 hours, whereas the percentage of dead cells increased severely to 40.1% and 53.7% for 182^R^-2 and 182^R^-2 cells, respectively. To investigate whether the induced cell death was caused by apoptosis, the apoptotic indicator PARP cleavage was measured by western blot analysis. Upon treatment for 96 hours with barasertib (50 nM), cleaved PARP (85 kDa) was seen in the resistant cells, whereas parental T47D cells only expressed full length PARP (116 kDa) (Figure 6C). This indicates that cell death induced by barasertib, at least in part, was caused by induction of apoptosis in the fulvestrant resistant T47D breast cancer cell lines.

### Aurora kinases are important for growth of tamoxifen resistant T47D cell lines

**Figure 7:**
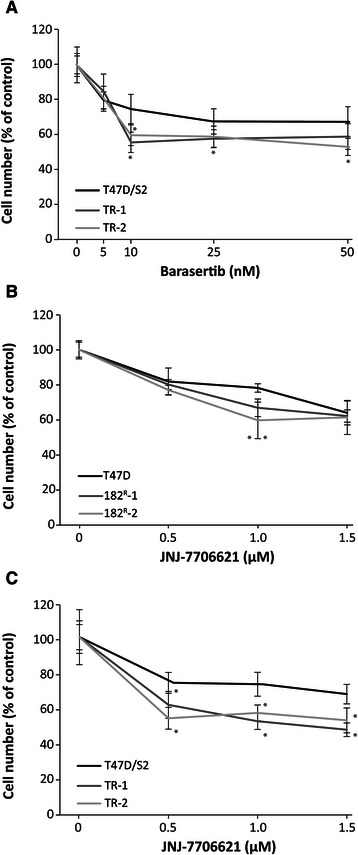
**Aurora kinases are important for growth of tamoxifen resistant cell lines.** Parental and tamoxifen resistant T47D cell lines (TR-1 and TR-2) were treated for 5 days with the indicated concentrations of barasertib **(A)** and parental, fulvestrant and tamoxifen resistant T47D cell lines were treated with JNJ-7706621 **(BandC)**. Cell number was determined by a crystal violet colorimetric assay and expressed as percent of untreated control. The experiments were performed twice with six sample replicates. Representative experiments with mean ± SD are shown. *P < 0.05 for barasertib or JNJ-7706621 treated samples vs. control.

To investigate if barasertib also inhibited growth of tamoxifen resistant cell lines, dose–response growth experiments with increasing concentrations of barasertib (5–50 nM) were conducted on the recently established tamoxifen resistant T47D breast cancer cell lines, TR-1 and TR-2 [28]. As seen in Figure 7A, treatment of the tamoxifen resistant T47D cells resulted in a statistically significant and preferential 40% growth inhibition at 10 nM barasertib compared to 30% growth inhibition of the parental T47D/S2. Thus, barasertib exerts preferential growth inhibition of both fulvestrant and tamoxifen resistant T47D breast cancer cell lines. The tamoxifen resistant T47D cell lines were less sensitive to barasertib than the fulvestrant resistant cell lines (Figure 1C). Ectopic expression of Aurora kinase A has recently been shown to confer resistance to tamoxifen in ER-positive MCF-7 breast cancer cells through phosphorylation of ERα [37], and as shown in Figure 7C, the Aurora kinase A/B inhibitor JNJ-7706621 exerted preferential growth inhibition of the ER-positive tamoxifen resistant T47D cell lines [28] compared to the parental T47D/S2 cells, whereas the ER-negative fulvestrant resistant cell lines [11] did not display preferential growth inhibition to JNJ-7706621, Figure 7B.

### Aurora kinase B as potential biomarker for disease-free and overall survival of tamoxifen treated breast cancer patients

**Figure 8:**
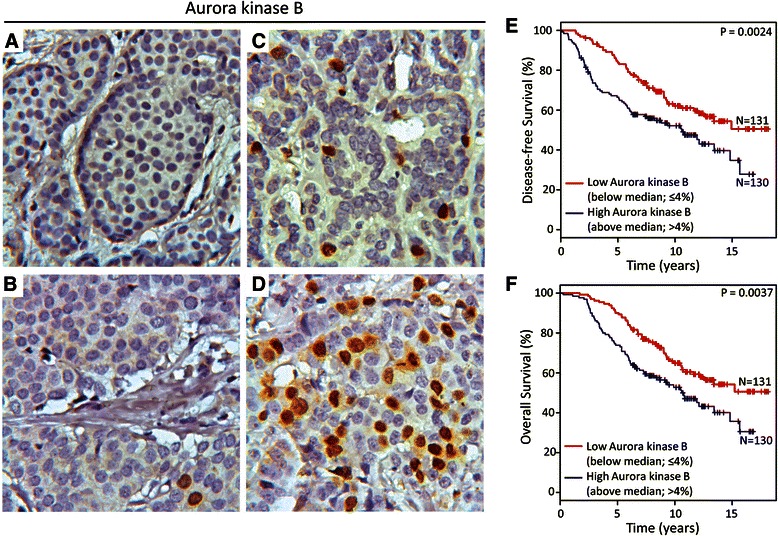
**High percentage of Aurora kinase B positive tumor cells in primary breast tumors is associated with reduced recurrence-free and overall survival. A-B**. Representative pictures showing negative/low percentage (≤4% positive cells, below median) of Aurora kinase B positive tumor cells. **C**-**D**. Representative pictures showing moderate and high percentage (>4% positive cells, above median) of Aurora kinase B positive tumor cells. Kaplan-Meier survival curves demonstrating percentage of **E)** disease-free and **F)** overall survival of patients with breast tumors showing low (≤4%) and high (>4%) percentage of Aurora kinase B positive tumor cells.

To investigate the association between Aurora kinase B protein expression and antiestrogen resistance, the expression of Aurora kinase B was evaluated in tumors from 261 high-risk ER-positive breast cancer patients, who had received tamoxifen as first-line adjuvant endocrine treatment. Aurora kinase B expression was confined to the nucleus of breast cancer cells and scored as percentage positive tumor cells (Figure 8A-D). Aurora kinase B was expressed in 259 (97.3%) of the tumor samples, in the range of 0-30% (Median 4%; interquartile range: 2–8.5%). Univariate analysis revealed a statistically significant association between the number of Aurora kinase B positive tumor cells and reduced disease-free and overall survival (P = 0.0067 and P = 0.0026, respectively). In multivariate analysis including the standard covariates; tumor grade, tumor size, nodal status and age, Aurora kinase B was not a significant and independent marker of decreased disease-free or overall survival. When the tumors were stratified into high (above median, >4%) or low (below median, ≤4%) percentage of Aurora kinase B positive cells, a significant association to disease-free (P = 0.0024) and overall (P = 0.0037) survival was seen (Figure 8E,F). Fifteen-years disease-free and overall survival for patients with high percentage of Aurora kinase B positive tumor cells was 30% compared to 50% for patients with low percentage of Aurora kinase B positive tumor cells (Figure 8E,F).

## Discussion

Although endocrine therapy targeting estrogen signaling through ER has clearly improved survival of breast cancer patients, treatment resistance is complex and a major clinical challenge. To explore the molecular mechanisms behind antiestrogen resistance, we have developed cell lines resistant to the antiestrogens fulvestrant and tamoxifen based on the estrogen responsive T47D breast cancer cell line, and utilized a kinase inhibitor screen to identify kinases involved in growth of the fulvestrant resistant T47D cell lines. We found that the Aurora kinase B specific inhibitor barasertib preferentially inhibited growth of the fulvestrant resistant T47D breast cancer cell lines compared with growth of the parental fulvestrant sensitive T47D cells. To verify the role of Aurora kinase B for fulvestrant resistant cell growth, siRNA-mediated knock-down experiments were performed with several siRNA constructs. However, we did not obtain significant knock-down with any of the constructs, including the construct which in our MCF-7 cells reduced Aurora kinase B expression [38]. Therefore, another Aurora kinase B specific inhibitor hesperadin was tested and the observed preferential growth inhibition of the fulvestrant resistant cell lines supports the role of Aurora kinase B for growth of fulvestrant resistant T47D cells. The inhibition with hesperadin at 1 μM was less pronounced than for 1 μM barasertib, which may be explained by the difference in potency of the two inhibitors, IC_50_ for barasertib is 0.37 nM and for hesperadin 250 nM (see www.selleckchem.com). Aurora kinase B is a key regulator of mitosis and essential for cell proliferation. It is the catalytic component of the chromosomal passenger complex (CPC), composed of Aurora kinase B, survivin, INCENP and borealin [22]. The complex is critical for accurate chromosomal segregation, cytokinesis, and regulation of the mitotic checkpoint [22]. We show that phosphorylation of Aurora kinase B and INCENP was increased in the fulvestrant resistant cell lines compared to the level in the parental T47D cells, indicating active CPC complex and suggesting that activation of Aurora kinase B protein is of particular importance for the fulvestrant resistant T47D cell lines. Over expression of Aurora kinase B has previously been found to interfere with chromosome bi-orientation and the spindle-assembly checkpoint due to enhanced disruption of kinetochore-microtubule attachments and sister chromatid cohesion [39]. In addition, Aurora kinase B over expression also caused abnormal cytokinesis resulting in chromosome segregation errors [39,40]. Although we do not find overexpression of Aurora kinase B in the fulvestrant resistant cell lines, our finding of increased level of the active form of Aurora kinase B and the active form of the downstream targets INCENP and mitosis specific histone H3, indicates that Aurora kinase B plays a major role for fulvestrant resistant cell growth, and the increased cell death, as measured by SYTOX-positive cells in the resistant cell lines, compared to the untreated parental T47D cell line, could possibly be caused by impaired cytokinesis.

In this study, barasertib preferentially inhibited fulvestrant resistant cell growth and phosphorylation of both Aurora kinase B and its targets INCENP and mitosis specific histone H3 in the resistant cells. Moreover, barasertib obstructed proper chromosome segregation and induced arrest of the fulvestrant resistant cells in the G2-phase. The increased proportion of cells in SubG1 together with PARP cleavage in the fulvestrant resistant cell lines indicates that barasertib-induced cell death is mediated by the apoptotic death pathway, as previously described in a panel of human myeloma cell lines [41]. Parental T47D cells treated with barasertib also displayed erroneous chromosome segregation and multinucleated cells. However, in contrast to the resistant cells, progression through the cell cycle was not prevented, rather the cells reentered a new cell cycle without cytokinesis, resulting in polyploid cells (>4N) and cell survival, at least for a period. Collectively, our results show that the fulvestrant resistant cells are more vulnerable to disturbance of proper cell cycle progression induced by treatment with barasertib, and suggest that the fulvestrant resistant cells are more dependent of Aurora kinase B for survival, resulting in cell death upon inhibition of Aurora kinase B. The primary goal of the study was to disclose the growth promoting pathways in fulvestrant resistant T47D cells. However; we also found significant and preferential growth inhibition of two tamoxifen resistant T47D cell lines with barasertib, but the effect was less pronounced in the tamoxifen resistant T47D cell lines compared to fulvestrant resistant cell lines. We have recently shown that Aurora kinase A and ER are major players in growth of tamoxifen resistant cell lines, including the tamoxifen resistant T47D cell lines [28], and Aurora kinase A has been found to confer tamoxifen resistance by activating ER by phosphorylation [37]. Thus, whereas Aurora kinase B appears to play a major role in the ER-negative fulvestrant resistant T47D cell lines [11], Aurora kinase B may play a minor role in the ER-positive tamoxifen resistant T47D cell lines. Major importance of Aurora kinase A has also been found in ER-positive aromatase inhibitor resistant cell lines, and Aurora kinase B also contributes to aromatase inhibitor resistant cell growth [38]. These data support the importance of Aurora kinases for growth of endocrine resistant breast cancer cells and whereas Aurora kinase B has a major role in ER-negative cells, Aurora kinase A appears to have a major role in ER-positive breast cancer cells.

Administration of barasertib has been shown to potently inhibit growth of colon, lung, hematologic and breast tumor xenografts as well as a panel of human breast cancer cell lines [35,42,43]. In clinical studies, no objective tumor response was observed in any of the patients with solid malignant tumors treated with barasertib. However, they generally tolerated barasertib well, and 23-25% of the barasertib-treated patients achieved prolonged disease stabilization [44,45] (see www.clinicaltrials.gov). Barasertib was also well tolerated in patients with acute myeloid leukemia enrolled in clinical phase I and I/II studies. These trials showed an overall response rate of 19-25% [46,47]. Collectively, these findings suggest that breast cancer patients resistant to antiestrogens could benefit from treatment targeting Aurora kinase B, e.g. barasertib.

At present, we do not know the mechanisms whereby the activity of Aurora kinase B is up regulated in our fulvestrant resistant cell lines or how Aurora kinase B is involved in resistant cell growth. The findings in this study suggest that the protein harbors key functions needed for the resistant cell lines to survive upon development of resistance. Although we do not find over expression of Aurora kinase B in the fulvestrant resistant T47D cell lines, over expression of Aurora kinase B may be important for tumor cell growth. Noteworthy, over expression of Aurora kinase B in Chinese hamster embryonic diploid fibroblasts results in aneuploidy cells capable of forming aggressive tumors in nude mice [40] and Aurora kinase B is found over expressed in several solid cancers, including breast, colorectal, kidney, lung, and prostate cancer [27]. Additionally, a correlation between Aurora kinase B expression and poor survival has been demonstrated in several cancers including glioblastomas, head and neck squamous cell cancer and lung cancer [27,48,49]. Here, immunohistochemical analysis performed on primary tumors from ER-positive breast cancer patients, receiving first-line adjuvant endocrine therapy with tamoxifen, revealed that high percentage (above median) of Aurora kinase B positive tumor cells was a marker for reduced disease-free and overall survival in this patient cohort. Thus, Aurora kinase B may be a marker for resistance to antiestrogens. However, since Aurora kinase B is an important cell cycle regulator, we cannot exclude that it is a proliferation marker, as resistant cells may have high proliferative activity. This is to the best of our knowledge the first study showing a link between high Aurora kinase B and reduced benefit from tamoxifen treatment. Based on our *in vitro* studies with the antiestrogen resistant T47D breast cancer cell lines, analysis of the association between Aurora kinase B and survival of breast cancer patients treated with fulvestrant would also be of great interest. Archival breast cancer tissue from such patients is unfortunately not available for us. Our previous findings of the importance of Aurora kinase A and ER for growth of tamoxifen and aromatase inhibitor resistant breast cancer cell lines [28,38] and for high Aurora kinase A expression as a marker for reduced response to tamoxifen therapy [28], indicate that both Aurora kinase A and B may be useful markers in endocrine resistant breast cancer and also targets for treatment.

## Conclusion

In this study we found that Aurora kinase B is an important kinase for growth and signaling in the ER-negative fulvestrant resistant T47D breast cancer cell lines and that Aurora kinase B also plays a role for growth of ER-positive tamoxifen resistant T47D cell lines. Aurora kinase B was identified as a biomarker for reduced benefit of tamoxifen treatment. Therefore, our results indicate that Aurora kinase B is a driving factor for growth of antiestrogen resistant T47D breast cancer cell lines, and a biomarker for reduced benefit of tamoxifen treatment. Thus, inhibition of Aurora kinase B, e.g. with the highly selective kinase inhibitor barasertib, could be a candidate new treatment for breast cancer patients with acquired resistance to antiestrogens.

## Abbreviations

Abl: Abelson
CDK: Cyclin-dependent kinases
Chk: Checkpoint kinase
CK: Casein kinase
CPC: Chromosomal passenger complex
DAB: 3.3-diaminobenzidine tetrahydrochloride
DBCG: Danish Breast Cancer Cooperative Group
ER: Estrogen receptor alpha
FAK: Focal adhesion kinase, FCS, fetal calf serum, FGFR, fibroblast growth factor receptor
Flt: Fms-like tyrosine kinase
IHC: Immunohistochemistry
INCENP: Inner centromere protein
JAK: Janus kinase
PDGFR: Platelet-derived growth factor receptor
PI3K: Phosphoinositide 3-kinase
Pyk: Proline-rich tyrosine kinase
SD: Standard deviation
SFKs: Src family kinases
TMA: Tissue micro arrays
VEGFR: Vascular endothelial growth factor receptor
